# My favourite flowering image: the remarkable inside-out flowers of *Lacandonia*

**DOI:** 10.1093/jxb/erae080

**Published:** 2024-05-20

**Authors:** Paula J Rudall

**Affiliations:** Jodrell Laboratory, Royal Botanic Gardens Kew, Richmond, Surrey TW9 3DS, UK

**Keywords:** Developmental constraints, flower evolution, morphological misfits, mycoheterotrophs

**Flowers are deceptively simple structures, characterized by a determinate primary axis that bears organs in condensed concentric zones in a strict structural and temporal sequence. Few species have escaped these constraints, but those that have can provide insights into the evolutionary history of flowers if placed in the appropriate phylogenetic and developmental context. For my flowering image, I selected a longitudinal section of a *Lacandonia* flower, which breaks a fundamental rule of spatial arrangement: the flowers are ‘inside-out’, with the carpels surrounding the stamens—a pattern that is almost unique among angiosperms. When viewed in the context of the family and order to which it belongs, this species has led me into many fascinating areas of comparative and evolutionary plant morphology.**

**Figure:**
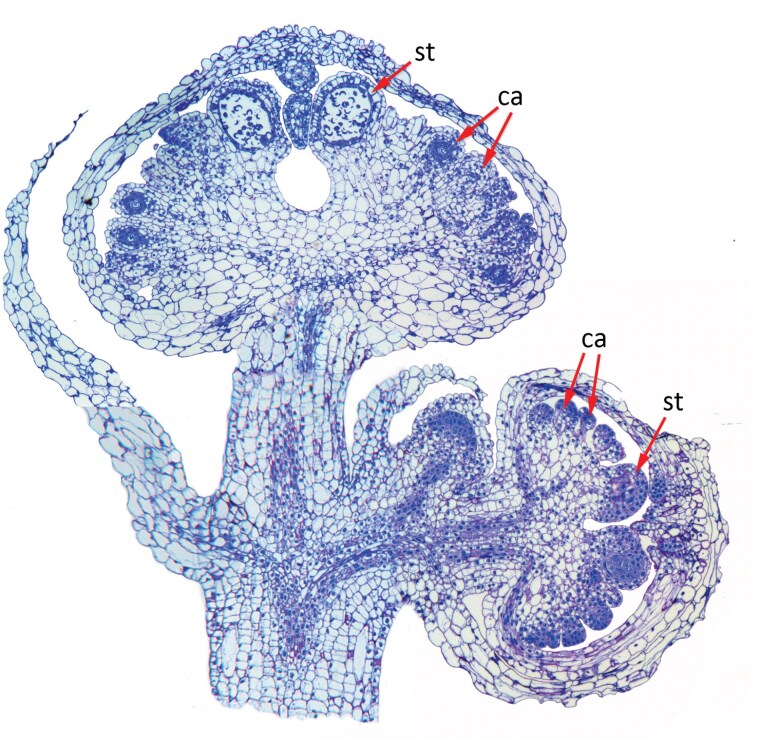
*Lacandonia brasiliana*, composite image of a longitudinal section of a flowering axis bearing three flowers at three contrasting developmental stages, showing the unique arrangement of multiple carpels (ca) surrounding stamens (st) in the centre of the flower.

Angiosperm families—and sometimes even entire orders—are often identifiable by particular floral features. The fact that we can readily recognize the characteristics of some major plant groups indicates that they are strongly canalized towards maintaining stable developmental programmes. For example, a relatively consistent defining feature of the eudicot clade, which encompasses >70% of extant angiosperm species, is the presence of a two-whorled pentamerous perianth, in which floral parts are arranged in alternating whorls of five organs each (Sauquet *et al.*, 2017). The pentamerous–pentacyclic condition is rare among non-eudicot angiosperms, highlighting its significance in terms of developmental regulation. In contrast, monocots typically display a trimerous–pentacyclic condition, with floral parts borne in threes (Remizowa *et al.*, 2010).

In a typical angiosperm flower, an outer region of sterile organs (the perianth) surrounds the pollen-bearing organs (the stamens), which in turn surround the distal/central ovulate organs (the carpels). At least in model eudicots such as *Arabidopsis*, the genetic basis for this organ arrangement was elegantly outlined in the iconic ABC model of floral development, stimulating many new studies of the dynamics of floral structure and development (Coen and Meyerowitz, 1991; Bowman and Moyroud, 2024). There is surprisingly little divergence from this pattern among angiosperms, suggesting an unusually strong developmental constraint. A notable exception to this rule is the monocot genus *Lacandonia*, in which the reproductive arrangement is reversed: several carpels surround three central early-formed stamens (Vergara-Silva *et al.*, 2003; Ambrose *et al.*, 2006). The inverted floral patterning of *Lacandonia* is almost unique among angiosperms, and has led some authors to question the flower–inflorescence boundary in this species (Rudall, 2003, 2017). Expression patterns of B-class and C-class floral homeotic genes are similarly inverted in the *L. schismatica* flower (Álvarez-Buylla *et al.*, 2010); this observation highlights one of the inevitable limits of the ABC model, which nicely explains the expression patterns of genes along a developing floral axis, but cannot determine the nature or evolutionary origin of the axis itself.

Triuridaceae belong to the ‘misfit’ monocot order Pandanales, in which several other taxa are highly anomalous, lacking structures that could readily be termed ‘true’ flowers; examples include *Cyclanthus* and *Sararanga*. This unusual collection of misfits indicates an evolutionary loss of developmental homeostasis within Pandanales at the phylogenetic node located immediately above Velloziaceae (Rudall and Bateman, 2006). Comparison of *Lacandonia* with other Triuridaceae is hampered by the lack of a well-resolved phylogenetic context, not only reflecting the complexity of evolutionary process but also resulting from a current paucity of molecular data and inadequate species sampling in this group. However, existing comparative morphological data indicate that the *Lacandonia* flower probably evolved from a structure with more typical organ patterning (e.g. Nuraliev *et al.*, 2020). The flowers of most other Triuridaceae are unisexual, although a few species (e.g. *Triuris brevistylis*) produce more conventional bisexual flowers in which stamen–carpel arrangement is not inside-out (Rudall, 2003, 2008; Espinosa-Matías *et al.*, 2012).

For many years, *Lacandonia* was known from only a single species, *L. schismatica* (Martínez and Ramos, 1989), which is confined to rare and scattered populations in the remote Lacandon rainforest of southeastern Mexico. However, more recently, a new species of *Lacandonia* was discovered by Brazilian botanists in northeastern Brazil, several thousand kilometres south of the first species; they described this second species as *L. brasiliana* (Melo and Alves, 2012). This new discovery prompted my collaborators and I to study the flowers in more detail, as part of a series of comparative studies of Pandanales (Rudall *et al.*, 2016). Both species of *Lacandonia* occur in rare and scattered populations, leading us to speculate whether this strange form had evolved only once (presumably followed by long-distance dispersal), or *in situ* from two independent mutations that became stabilized to form two new species. Our study showed that the two species of *Lacandonia* are indeed remarkably alike in morphology, despite the large geographical separation between them. Their strong similarity suggests that they could have resulted from a single mutation, though this inference is difficult to demonstrate conclusively. Both species have inside-out flowers with three central stamens surrounded by many carpels; the carpels themselves are arranged on ridges in radial double rows, termed fascicles, which represents another highly unusual feature in angiosperms. In both species, the anthers remain closed and do not shed their pollen in the normal way (Márquez-Guzmán *et al.*, 1993; Rudall *et al.*, 2016). Amazingly, in both species, fertilization and seed set are apparently achieved by pollen grains germinating within the anther and growing through the tissues of the same plant to the female organs—a rare phenomenon that has been reported in only a few other flowering plants, such as the monocot *Sagittaria potamogetifolia* (Wang *et al.*, 2002).

In common with all other species of Triuridaceae, *Lacandonia* is mycoheterotrophic. Mycoheterotrophs are translucent-yellow rather than green because their chlorophyll is highly reduced or absent, so they are unable to produce carbohydrates by photosynthesis; instead, they rely on soil fungi for nutrition. Therefore, *Lacandonia* lacks at least two of the most important factors that govern plant structure: typical floral patterning and chlorophyll-mediated photosynthesis. Can this remarkable combination of deviations from the norm be a coincidence? Long ago, the botanist William Hemsley (1843–1924), former Keeper of the Kew Herbarium and a great friend of Charles Darwin, commented on the family Triuridaceae in general that ‘it would appear that these small flowers are peculiarly subject to disturbances in their development’ (Hemsley, 1907). Perhaps the fact that Triuridaceae are mycoheterotrophs has relaxed evolutionary–developmental constraints sufficiently to permit the inside-out flowers of *Lacandonia*. A recent transcriptomic analysis demonstrated convergent evolutionary changes in expressed nuclear genes from three independent mycoheterotrophic monocot lineages (Timilsena *et al.*, 2023). Although these results remain inconclusive, they highlight the possibility of convergent evolution associated with gene loss.

However, other processes can also cause species to overcome strong developmental constraints and result in the evolution of novel growth forms. For example, terminal flower-like structures in racemose inflorescences can result in secondary derivation of flower-like structures, as in the early-divergent monocot order Alismatales (Sokoloff *et al*., 2006). The resulting overlap in expression zones of key regulatory genes could lead to morphological novelties. The only other living angiosperm with inside-out reproductive units is the water-lily relative *Trithuria* (family Hydatellaceae), which is an ephemeral aquatic that is not mycoheterotrophic. As in *Lacandonia*, debate regarding homology has focused on the flower–inflorescence boundary in Hydatellaceae, a family that is placed phylogenetically close to the basal node of the angiosperm tree. The *Trithuria* reproductive unit, with its condensed inside-out structure, could represent an inflorescence-like structure derived from a secondarily modified flower (Rudall *et al.*, 2009).

It appears impossible to resolve conclusively evolutionary questions about the flower merely by comparing living structures, even when taken in the context of corresponding developmental–genetic and phylogenetic data. Comparative data, both morphological and molecular, often provide conflicting evidence. However, these studies illustrate that much remains to be explored in floral evolution. Studies of *Lacandonia* and other such ‘misfit’ plants provide us with greater insights into what precisely are the rules that govern plant structure, and how they may periodically be broken.
